# A systematic review comparing the evidence for kidney function outcomes between oral antidiabetic drugs for type 2 diabetes

**DOI:** 10.12688/wellcomeopenres.14660.1

**Published:** 2018-06-19

**Authors:** Samantha V. Wilkinson, Laurie A. Tomlinson, Masao Iwagami, Heide A. Stirnadel-Farrant, Liam Smeeth, Ian Douglas

**Affiliations:** 1 Department of Non-Communicable Disease Epidemiology, London School of Hygiene and Tropical Medicine, London, WC1E 7HT, UK; 2 RWD & Epidemiology, GSK R&D, Stevenage, SG1 2NY, UK

**Keywords:** Review, Kidney Diseases, Comparative Effectiveness Research, Diabetes Mellitus, Type 2, Hypoglycemic Agents

## Abstract

**Background**: The development of kidney disease is a serious complication among people with type 2 diabetes mellitus, associated with substantially increased morbidity and mortality.  We aimed to summarise the current evidence for the relationship between treatments for type 2 diabetes and long-term kidney outcomes, by conducting a systematic search and review of relevant studies.

**Methods**: We searched Medline, Embase and Web of Science, between 1st January 1980 and 15th May 2018 for published clinical trials and observational studies comparing two or more classes of oral therapy for type 2 diabetes. We included people receiving oral antidiabetic drugs. Studies were eligible that; (i) compared two or more classes of oral therapy for type 2 diabetes; (ii) reported kidney outcomes as primary or secondary outcomes; (iii) included more than 100 participants; and (iv) followed up participants for 48 weeks or more. Kidney-related outcome measures included were Incidence of chronic kidney disease, reduced eGFR, increased creatinine, ‘micro’ and ‘macro’ albuminuria.

**Results:** We identified 15 eligible studies, seven of which were randomised controlled trials and eight were observational studies. Reporting of specific renal outcomes varied widely. Due to variability of comparisons and outcomes meta-analysis was not possible. The majority of comparisons between treatment with metformin or sulfonylurea indicated that metformin was associated with better renal outcomes. Little evidence was available for recently introduced treatments or commonly prescribed combination therapies.

**Conclusions**: Comparative evidence for the effect of treatments for type 2 diabetes on renal outcomes, either as monotherapy or in combination is sparse.

## Introduction

Type 2 diabetes mellitus (DM) increases an individual’s risk for health problems including cardiovascular disease, blindness, chronic kidney disease (CKD), and nerve damage ^1– 4^. The development of kidney disease is associated with other complications of type 2 diabetes and with poorer outcomes ^1, 3, 5^. Therefore, slowing the development of, or preventing kidney disease is one aim of therapy ^2^. Type 2 diabetes drugs are thought to play a major role in protecting the kidneys by controlling blood sugar levels and may confer additional protective effects according to specific drug profiles ^3^. However, as kidney function declines, type 2 diabetes drug options become limited due to prescribing restrictions ^2, 3, 5– 7^. This presents a challenge for treating type 2 diabetes in patients with non-diabetic related kidney disease, as well as those with renal diabetic complications.

Treatment choice reflects a complex balancing of expected risks and benefits. A recent systematic review focused on vascular outcomes, glyclated hemoglobin (HbA1c), body weight, hypoglycaemia and common adverse events ^8^. Here we focus on kidney-related outcomes as another important aspect of clinical care that clinicians must consider when prescribing drugs for type 2 DM. Our aim was to provide a summary of the current evidence of long term kidney outcomes, from comparative, long terms studies of oral antidiabetic drugs. We included the following outcomes: change in kidney function (estimated glomerular filtration rate), progression or development of proteinuria, development of end-stage renal disease (ESRD) and composite outcomes compared between different oral drugs for the treatment of type 2 DM.

## Methods

The protocol for this systematic review was submitted, reviewed and approved by PROSPERO (International prospective register of systematic reviews, ref. 2016: CRD42016036646). The study was conducted and is reported in accordance with the PRISMA protocol ( Supplementary File 1) ^9^.

### Data sources and searches

We searched the databases; Medline, Embase and Web of Science for articles published between 1 ^st^ January 1980 and 15 ^th^ May 2018. The search comprised keywords and MESH terms relating to three broad themes: kidney function, type 2 diabetes drugs and clinical studies. We limited the search to English-language studies, and studies in humans. The search strategies are in Supplementary Table 1 and Supplementary Table 2 ( Supplementary File 2). The reference lists of relevant reviews identified through the search were also screened.

### Study selection

One reviewer (SW) screened all citations identified in the searches. Titles and abstracts for all studies were compared to the selection criteria. Then the full-text of selected studies were reviewed against the inclusion and exclusion criteria. Reviewer two (MI) was blinded to the articles selected by reviewer one and screened a 20% sample of the articles selected by reviewer one after the title screen. The studies chosen by the two reviewers were compared.

We defined the search and screening strategies before completing the searches. Studies were eligible for inclusion if they were clinical studies that (i) compared two or more classes of oral therapy for type 2 DM; (ii) reported kidney outcomes as primary or secondary outcomes; (iii) included more than 100 participants, and (iv) followed participants for 48 weeks or more. We restricted the review to oral antidiabetic drugs recommended at the initiation and first intensification of treatment ^6^.

We did not include studies that reported only placebo-controlled comparisons as we were interested in the difference in effects between active therapy regimes to reflect therapy choices made in routine clinical care; placebo-controlled studies would not estimate this difference. Our definition of a kidney outcome was broad to identify as many studies as possible. We accepted any kidney-related outcome, including the incidence of chronic kidney disease, reduced estimated Glomerular Filtration Rate (eGFR), increased creatinine, ‘micro’ and ‘macro’ albuminuria, proteinuria, end stage renal disease (ESRD) and composite kidney outcomes. We did not include composite microvascular outcomes that combined kidney outcomes with other microvascular outcomes such as retinopathy or neuropathy.

### Data extraction and quality assessment

After study selection, using a predefined data collection tool, we extracted data for the following items: number of participants, study design, calendar years covered by the study, length of follow-up, drug comparison, mean age of study population, exclusion criteria for study, kidney measurements taken at baseline, mean duration of diabetes, mean HbA1c at baseline, primary outcome for the study, kidney outcomes reported and results for kidney outcomes reported. Reviewer one (SW) assessed each study for quality, using the GRACE 2014 ^10^ items for observational comparative effectiveness research and the Cochrane Collaboration tool for assessing risk of bias in randomised trials ^11^ for RCTs.

## Results

**Figure 1.**
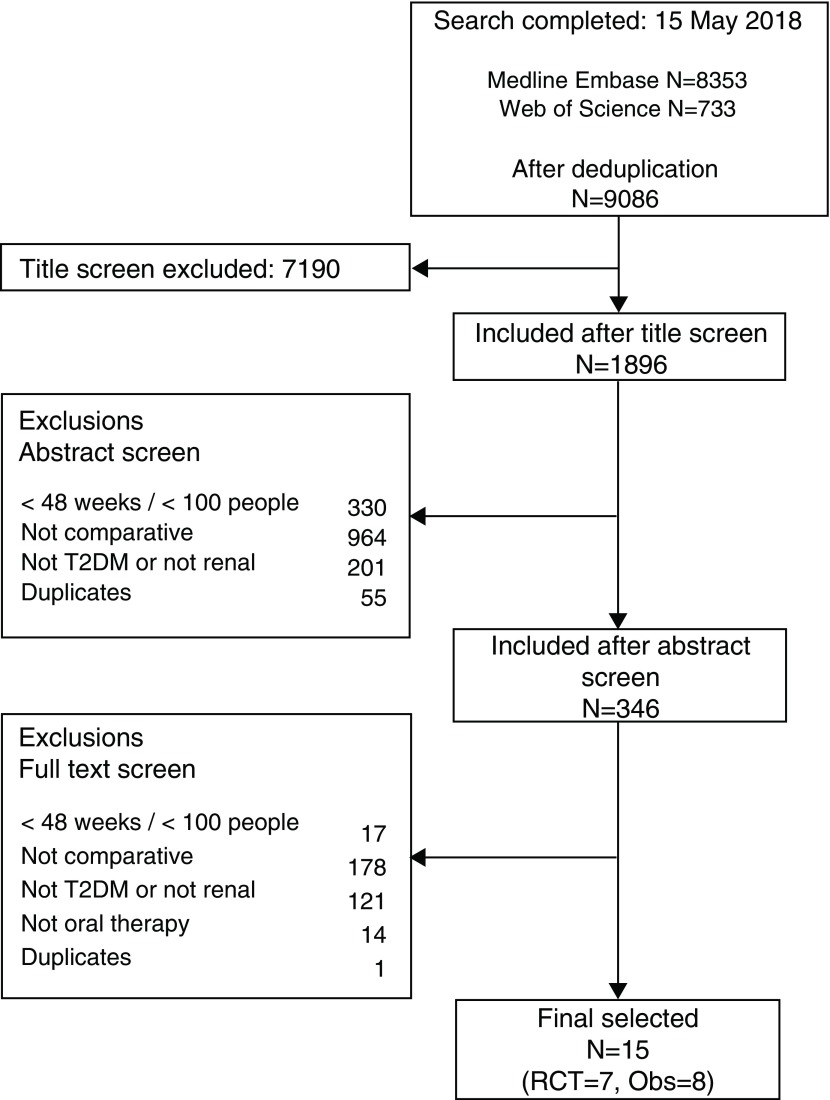
Flow diagram of study selection. Ovid was used to search the Embase and Medline databases.

Figure 1 details the study selection process through which we found 9,086 potentially eligible studies. The first reviewer (SW) completed the initial title screen and selected 1,896 articles. The second reviewer (MI) was blinded and reviewed a 20% random sample of these articles. The agreement between reviewers was good, reviewer two selected an additional paper that was rejected after discussion. After subsequent discussions (SW, MI and LT), we selected 15 studies.

**Figure 2.**
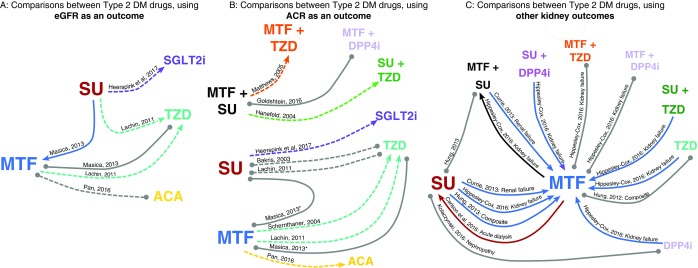
Graphical representation of drug comparisons and findings. Connecting lines indicate where studies have made comparisons between drugs. Lines connect drug names and are labelled with the authors that made the comparison. Dashed line indicates randomised studies, single line indicates non-interventional studies. Findings are indicated by the colour of the line: where one drug appears to be protective, the line is the colour of the protective drug. Grey lines indicate no significant difference. E.g. Blue lines connecting metformin to sulfonylurea indicate that metformin appeared to be protective of kidney function. Arrow heads point towards the drug that appeared to be protective. One further comparison not included here. Hung *et al.* 2012, as two studies by Hung *et al.* reported similar comparison using similar data* Also includes dipstick and urine protein tests, † metformin group largely metformin, but some taking TZD or SU. Abbreviations: MTF: metformin, SU: sulfonylurea, TZD: Thiazolidinedione, DPP4i: Dipeptidyl peptidase-4 inhibitor, ACA: acarbose, SGLT: Sodium-glucose Cotransporter 2 inhibitors, GLP1: Glucagon-like peptide-1 receptor agonist, eGFR: estimated Glomerular Filtration Rate, ACR: Albumin creatinine ratio, ARF: Acute renal failure.

We identified 15 eligible studies, seven of which were randomised controlled trials (RCTs) ^12– 18^ and eight were observational studies ^19– 26^. Across the 15 studies, three RCTs ^16– 18^ and one observational study ^22^, reported changes in eGFR as an outcome. All seven RCTs ^12– 18^ and two observational studies ^22, 25^ investigated albumin-creatinine ratio (ACR) as an outcome. Six observational studies reported kidney endpoints, including kidney failure, nephropathy, acute dialysis and composite endpoints with eGFR ^19– 21, 23, 24, 26^. Comparisons made, and outcomes studied are summarised graphically in Figure 2. Given the range of the kidney function outcomes reported and the drug class comparisons made we did not complete a meta-analysis of the results, instead we provide a narrative summary of studies. Selected studies and their findings are summarised in Table 1 and Table 2.

**Table 1.** Summary of study characteristics: Randomised Studies.

Author (Year)	Number	Follow- up	Drug comparison ^*^	Mean age (yrs)	Exclusions ^†^	Inclusions ^†^	Measures at baseline			Primary outcomes of study	Kidney outcomes recorded
							Kidney measures Proteinuria/ Mean ACR/ eGFR	Yrs with T2DM Mean (SD)	Mean HbA1c(%, SD)		
Bakris *et al* (2003) ^12^	121 ^a^	52w	SU, TZD (GLY, RSG)	55.6	Prior use of ACEI, ARBs, BB or CCBs	40–80 yrs with type 2 DM	28% micro- albuminuria ^b^ Baseline ACR NR	NR	GLY: 9.5 (1.6) RSG: 9.1 (1.7)	Change in left ventricular mass index	52 w Microalbuminuria ^b^ resolved in: RSG: 43%, GLY: 6% ACR mean % change: RSG: -23, GLY: -8
Hanefeld *et al* (2004) ^13^	639	52w	SU+TZD, SU+MTF (SU+PGZ, SU+MTF)	60	Previous cardiac events, malignant disease in 6 months before study. Previous treatment with MTF or TZD	35–75yrs with type 2 diabetes inadequately managed with SU monotherapy with HbA1c 7.5-11.0%	28% albuminuria ^c^ Mean ACR (SD) SU+PGZ: 0.07 (0.25) SU+MTF: 0.11(0.56)	7	SU+PGZ: 8.8 (0.98) SU+MTF: 8.8 (0.97)	HbA1c at week 52, FPG, Insulin and lipid profiles.	52 w Microalbuminuria ^c^ resolved in: SU+ PGZ: 10.2%, SU+MTF: 7.7% ACR mean % change: SU+ PGZ: -15, SU+MTF: +2
Schernthaner *et al* (2004) ^14^	1199	12m	MTF, TZD ( MTF, PGZ )	56.5	Use of thiazides but other antihypertensives allowed	People inadequately treated with di *et al*one, or HbA1c 7.5–11%	NR	3.3	PGZ: 8.7 (1) MTF: 8.7 (1)	HbA1c	52 w ACR mean % change: PGZ: -19, MTF -1
Matthews *et al* (2005) ^15^	630	52w	MTF+TZD, MTF+SU ( MTF+PGZ, MTF+GLZ )	56.5	Ketoacidosis, MI, TIA, stroke in the previous 6m; symptomatic heart failure; acute malabsorption or chronic pancreatitis; familial polyposis coli; malignant disease in past 10ys; substance abuse	Previously not managed with MTF monotherapy, HbA1c 7.5–11%. No previous treatment with insulin, gliclazide, pioglitazone, SU/ TZD	Mean ACR (SD) MTF+PGZ: 0.06 (0.14) MTF+GLZ: 0.05(0.16)	5.7	SU+Pio: 8.7 (0.1) SU+MTF: 8.53 (0.9)	HbA1c	52 w ACR mean % change: MTF+ PGZ: -10, MTF+GLZ: +6
ADOPT Lachin *et al* (2011) ^16^	4351	5yrs	TZD, MTF, SU (RSG, MTF, GLY)	56.9	Significant liver disease, kidney impairment (serum creatinine males: >1.3mg, females: >1.2mg), history of lactic acidosis, angina, congestive heart failure uncontrolled hypertension	≥3yrs history of type 2 DM, FPG 7-10mmol/L.	16% albuminuria ^c^ Mean ACR (log transformed) RSG 9.9 (180), MTF 9.3 (172), GLY 9.4 (172) Mean eGFR (geometric): RSG 98.0 (24.6), MTF 97.1 (25.6), GLY 95.7 (27.6)		RSG: 7.36 (0.93) MTF: 7.36 (0.93) GLY: 7.35 (0.92)	Time to drug failure, using FPG	4 yr Albuminuriad resolved in: RSG: 69.5%, MTF: 64%, GLY: 64% ACR mean change (95% CI): RSG 2.1 (-4.2, 8.8), MTF 20.9 (13.3, 28.9), GLY 6.1 (-1.2, 14.0) eGFR mean change % (95% CI): RSG: 5.1 (3.6-6.7), MTF: 1.4 (0.0, 2.9), GLY: -0.4 (-2, 1.2)
Pan *et al* (2016) ^18^	762	48w	ACA, MTF	50	History of cardiac disease, kidney disease, uncontrolled hypertension, urinary infection	Newly diagnosed type 2 diabetes within 1 yr: >1 month of treatment with type 2 diabetes in previous 12m and no treatment 3 months prior.	Elevated ACRe ACA 20%, MTF 24% Median ACR (IQR) ACA: 12.5 (4.9- 25.8), MTF 11.6 (5.3-28.8) Mean eGFR (SD) ACA: 109.6 (29.8), MTF 114.9 (32.3)	ACA: 1.6, MTF: 1.7	ACA: 7.49 (1.25) MTF: 7.6 (1.23)	ACR, eGFR	Elevated ACRe Median ACR (IQR) ACA: 5.80 (0.9-13.2), MTF 7.31 (2.2-18.7) Mean eGFR (SD): ACA: 112.8 (32.6), MTF 114.6 (32.8)
CANTATA-SU Heerspink *et al* (2017) ^17^	1450	104w	SGLT, SU (CNG, GLM)	56.2	eGFR >60, last 6 months severe hypoglycaemia, serum creatinine (μmol/L) (men >124, women >115), TZD in last 16 weeks	18-80 yrs with type 2 DM, HbA1c 7-9.5 %. managed with MTF therapy	Mean ACR (25th, 75), CNG 100mg: -2.7 (-3.5, -1.9), CNG 300mg: percentile) GLM: 8.2 (5.75, 17.98), CNG 100mg: 8.7 (5.74, 17.52), CNG 300mg: 8.6 (5.28, 20.64) Mean eGFR (SD) GLM: 89.5 (17.5), CNG 100mg: 89.7 (19.3), CNG 300mg: 91.4 (19.4)	6.6	GLM: 7.8 (0.8) CNG 100mg: 7.8 (0.8) CNG 300mg: 7.8 (0.8)	Change in albuminuria and kidney function	104w ACR mean % change, relative to GLM (SD): CNG 100mg: -5.7 (2.2, -13.1), CNG 300mg: -11.2 (-3.6, -18.3) eGFR Mean change (95 CI): GLM: -5.4 (-6.2, -4.5), CNG 100mg: -2.7 (-3.5, -1.9), CNG 300mg: -3.9 (-4.7, -3.0) Incidence of 30% eGFR decline HR (95% CI) Referent GLM CNG 100mg: 0.66 (0.42, 1.04), CNG 300mg:0.93 (0.62, 1.42)

**Abbreviations**: ACA: acarbose, ACEI: ACE Inhibitor, ACR: Albumin:Creatinine Ratio, ARB: Angiotensin receptor blocker, BB: beta-blocker, CCB: calcium channel blocker, CI: confidence interval, CNG: Canagliflozin, CV: coefficient of variation [100x(exp[SD-mean])], eGFR: estimated glomerular filtration rate, FPG: Fasting plasma glucose, GLY: glyburide, GLZ: Gliclazide, GLM: Glimepiride, IQR: Inter Quartile Range, MI myocardial infarction, MTF: metformin, NR: not reported , PGZ: Pioglitazone, RSG: Rosiglitazone, SU: sulfonylurea, SGLT: SGLT2i, SD: Standard deviation, TZD: thiazolidinedione, TIA: transient ischaemic attack

Notes: ^*^Oral type 2 diabetes drugs only, ^†^Summary inclusion and exclusion criteria only, a: N with ACR at baseline and by 52w, b: Defined as ACR 30 µg/mg or below [or 30mg/g], c: Not defined, d: ACR greater than or equal to 30mg/g, e: elevated ACR included ‘micro’ albuminuria (30-300mg/g) and ‘macro’ albuminuria (≥300mg/g)

**Table 2.** Summary of study characteristics: Observational Studies.

Author (Year)	Number	Data source (Country)	Yrs of study	Drug comparison	Age (yrs)	Kidney related exclusions	Measures at baseline			Primary outcomes of study	Follow-up (yrs)	Kidney outcomes recorded HR (95% CI) ^c^
							Kidney	Years with T2DM	HbA1c %			
Hung *et al.* (2012) ^19^	93577	Veterans Administration (US)	2001– 2008	Incident MTF, SU or RSG, excluding combination users	Median (IQR) MTF: 60 (55, 69) SU: 62 (56, 72) RSG: 64 (57, 72)	eGFR <60	Microalbuminuria ^b^ %: MTF: 3, SU: 3, RSG: 4 [only available for 15,065 people] Median eGFR (IQR) MTF: 81 (72, 93), SU: 80 (70, 93), RSG: 79 (69, 91)	NR	Median (IQR): MTF: 7.1 (6.5, 7.9) SU: 7.3 (6.6, 8.4) RSG: 6.8 (6.2, 7.6)	**1** eGFR (≥25% decline) **2** ESRD (eGFR<15, ICD-9 codes for dialysis or renal transplant) **3** Mortality	Median (IQR): MTF: 0.9 (0.5, 1.8) SU: 0.8 (0.4, 1.7) RSG: 0.7 (0.3, 1.5)	**eGFR event** **or ESRD** Referent MTF SU: 1.20, (1.13, 1.28), RSG: 0.92, (0.71, 1.18) **eGFR event,** **ESRD or** **mortality** Referent MTF: SU 1.20, (1.13, 1.28), RSG: 0.89, (0.69, 1.12)
Currie *et al.* (2013) ^21^	84,622	CPRD GOLD datalink (UK)	2000– 2010	MTF, SU, MTF+SU	Mean (median) 61.9 (12.8)	None stated	Creatinine >130 µmol/L: 4.5%	Mean: 2.3 (SD 3.0)	Mean (SD): 8.7 (1.9)	Renal failure (Read codes)	Mean: 2.8	**Renal failure** Referent: MTF SU: 2.63 (2.20, 3.15), MTF+SU: 1.39 (1.12, 1.72)
Hung *et al.* (2013) ^20^	13238	Veterans Administration (US)	1999– 2008	MTF, SU, MTF+ SU	Median (IQR) MTF: 59 (54, 67) SU: 60 (54, 71) MTF+SU: 58 (53, 65)	Serum creatinine >1.5 mg/dL or eGFR < 60	eGFR Median (IQR) MTF: 81 (72, 93) SU: 80 (71, 93) MTF+SU: 82 (73, 97)	NR	Median (IQR) MTF: 7.1 (6.5, 7.9) SU: 7.3 (6.6, 8.4) MTF+SU: 7.9 (6.8, 10)	**1** eGFR (≥25% decline) **2** ESRD (eGFR<15, ICD-9 codes for dialysis or renal transplant) **3** Mortality	Mean: 1.2	**eGFR event** **or ESRD** Referent: SU MTF: 0.85 (0.72, 1.01), SU+MTF: 1.01 (0.75, 1.37) **eGFR event,** **ESRD or** **mortality** Referent: SU MTF: 0.82 (0.70, 0.97), SU+MTF 1.05 (0.79, 1.40)
Masica *et al.* (2013) ^22^	Proteinuria analysis: N=798 eGFR analysis: N=977 [IPW cohort]	Clinical data from primary care networks (US)	1998– 2009	Exposure to drug (≥90d) MTF, SU, TZD, or combo	Mean (SD) MTF: 53.9 (11.9) SU: 53.7 (13.0) TZD: 53.9 (12.0) [Age at diagnosis, IPW cohort]	Baseline proteinuria or MDRD eGFR<60	eGFR Mean (SD) **Proteinuria** **analysis**: MTF: 82.3 (20) SU: 79.5 (23) TZD: 75.6 (16) **eGFR analysis**: MTF: 86.8 (18) SU: 86.2 (21) TZD: 91.4 (34)	NR	8.0 % IPW group	**1** New proteinuria (24-hour albumin/protein, spot protein, spot ACR, or dipstick) **2** New eGFR <60	Proteinuria analysis: Mean: 3.2 eGFR analysis: Mean: 2.8	9% (72/798) developed proteinuria **Incidence of** **proteinuria** MTF referent SU: 1.27 (0.93, 1.74), TZD: 1.00 (0.70, 1.42) **Fall in eGFR** **to <60 (2)** MTF referent SU: 1.41 (1.05, 1.91), TZD: 1.04 (0.71, 1.50)
Hippisley- Cox and Coupland (2016) ^23^	274,324 [N for kidney analysis not reported]	QResearch (UK)	2007 – 2015	DPP4i, TZD, MTF, SU, ‘other agents’	Mean (SD) TZD: 63 (12) DPP4I: 63 (12) MTF: 64 (13) SU: 66 (13) Other: 60 (12)	Kidney disease at baseline, and severe kidney disease	NR for kidney analysis: prior to kidney baseline exclusions: Creatinine µmol/L mean (SD) TZD: 87 (34), DPP4I: 85 (33), MTF: 85 (30), SU: 92 (48)	% 1–3yrs since diagnosis: TZD: 28 DPP4I: 26 MTF: 25 SU: 24	Mmol/mol Mean (SD) TZD: 67 (19) DPP4i: 68 (18) MTF: 61 (19) SU: 65 (20) Other: 71 (20)	Incident severe kidney failure (Read codes for dialysis & transplantation, or CKD stage 5 based on serum creatinine values)	NR	**Incident** **severe** **kidney failure** MTF referent TZD: 2.55 (1.13, 5.74), DPP4i: 3.52 (2.04, 6.07), SU: 2.63 (2.25, 3.06), MTF+SU: 0.76 (0.62, 0.92), MTF+TZD: 0.71 (0.33, 1.50), MTF+DPP4i: 0.59 (0.28, 1.25), SU+TZD: 2.14 (1.27, 3.61), SU+DPP4I: 3.21 (2.08, 4.93)
Kolaczynski *et al.* (2016) ^24^	5436 matched sample	IMS Lifelink (Germany)	2007– 2013	SU, DPP4i	Mean (SD) SU: 63.7 (10.7) DPP4I: 64.6 (10.9)	History of nephropathy	Renal failure % (ICD-10 code) DPP4I: 13 SU: 11.1	Mean (SD) DPP4I: 3.1 (3.4) SU: 3.2 (3.4)	Mean (SD) DPP4i: 7.61 (1.47), SU: 7.64 (1.37)	Incident nephropathy (ICD-10 code)	Mean (SD) DPP4I: 3.48 (3.75) SU: 2.49 (3.46)	**Incidence of** **nephropathy** Referent SU DPP4i 0.90 (0.72, 1.14)
Goldshtein *et al.* (2016) ^25^	564 matched sample	Maccabi Health Service diabetes registry (Israel)	2008– 2014	MTF+SU, MTF+DPP4i	Mean (SD) SU: 58.5 (11) DPP4I: 59.1 (11.2)	Dialysis, eGFR <45 or ACE/ARB in 90 day post index	ACR mg/g mean (SD) SU: 122.4 (194.5) DPP4I: 139.9 (261.9) eGFR mean (SD) SU: 84 (19.5), DPP4I: 82.4 (19.1)	Mean (SD) SU: 5 (3.5), DPP4I: 5.2 (3.5)	Mean (SD) SU: 8.6 (1.5), DPP4i: 8.5 (1.5)	Improvements in urinary ACR (≥20% improvement in ACR and change in KDIGO category)	Mean: 9 months, max 52 weeks	**ACR** **reductions** Referent MTF+SU MTF+DPP4i: 1.20 (0.99,1.47)
Carlson *et al.* (2016) ^26^	168,443	All Danish citizens	2000– 2012	MTF, SU	Mean (SD) MTF: 65.7 (9.4) SU: 69.2 (10.8)	ESRD or eGFR <30 ml/min/1.73m ^2^	eGFR Median (IQR) MTF: 74 (63–87) SU: 69 (57–82)	NR	NR	**1** Acute dialysis	1y following treatment initiation	**Acute** **dialysis** Referent: SU MTF: 1.51 (1.06–2.17)

**Abbreviations**: ACR: Albumin: Creatinine Ratio, eGFR: estimated glomerular filtration rate, ESRD: End Stage Renal Disease, ICD: International Classification of Diseases, MTF: metformin, SU: sulfonylurea, TZD: Thiazolidinedione, DPP4i: Dipeptidyl peptidase-4 inhibitor, RSG: Rosiglitazone, STG: Sitagliptin, EXE: Exenatide. IPW: Inverse Probability Weight, FU: Follow-up, SD: Standard deviation, ARF: Acute Renal Failure, CKD: Chronic Kidney Disease, IQR: Inter Quartile Range, p-yr: person-years, NR: Not reported, DB: Database, KDIGO: Kidney Disease: Improving Global Outcomes **Notes**: a: MACE: Major adverse cardiac event: non-fatal MI, non-fatal stroke, or cardiovascular death, b: microalbuminuria if ACR was >30 mg/g, c: Hazard Ratio (HR), Mantel Haenszel (MH) or Odds Ratio (OR), eGFR units: mL/min/1.73m ^2^

In total, we identified 32 direct comparisons between oral drugs for the treatment of type 2 DM: 22 comparisons between monotherapies, three comparisons between dual therapy combinations, and seven comparisons between dual therapies and monotherapies, outlined in Table 3. One study compared many combination therapy options to metformin; we did not include the triple therapy combinations from this study in our results, details of the comparisons are in Supplementary Table 3 ( Supplementary File 2) ^23^.

**Table 3.** Results summary.

			RCTs		Observational	
			**Number**	**Results**	**Number**	**Results**
**ACR**						
**Monotherapy**						
**MTF** vs **ACA**			**1**	**Favours ACA**	0	
**MTF** vs **SU**			0		1	No difference
**MTF** vs **TZD**			**2**	**Both favour TZD**	1	No difference
**SU** vs **SGLT**			**1**	**Favours SGLT**	0	
**SU** vs **TZD**			2	Both no difference	0	
**Dual therapy**						
**MTF+SU** vs **MTF+DPP4i**			0		1	No difference
**MTF+TZD** vs **MTF+SU**			**1**	**Favours MTF+TZD**	0	
**SU+TZD** vs **SU+MTF**			**1**	**Favours SU+TZD**	0	
**eGFR**						
**Monotherapy**						
**MTF** vs **ACA**			1	No difference	0	
**MTF** vs **SU**			0		**1**	**Favours MTF**
**MTF** vs **TZD**			**1**	**Favours TZD**	1	No difference
**SU** vs **SGLT**			**1**	**Favours SGLT**	0	
**SU** vs **TZD**			**1**	**Favours TZD**	0	
**KIDNEY** **OUTCOMES**						
**Monotherapy**						
**MTF** vs **DPP4i**			0		1	**Favours MTF**
**MTF** vs **SU**			0		**4**	**3 favour MTF, 1 favours SU**
**MTF** vs **TZD**			0		2	1 no difference, **1 favours MTF**
**SU** vs **DPP4i**			0		1	No difference
**Mono vs. dual therapy**						
**MTF** vs **MTF+DPP4i**			**0**		1	No difference
**MTF** vs **MTF+SU**			0		2	**1 favours MTF, 1 favours** **MTF+SU**
**MTF** vs **MTF+TZD**			0		1	No difference
**MTF** vs **SU+DPP4i**			0		**1**	**Favours MTF**
**MTF** vs **SU+TZD**			0		**1**	**Favours MTF**
**SU** vs **MTF+SU**			0		1	No difference

**Abbreviations**: ACR: Albumin: Creatinine Ratio, eGFR: estimated glomerular filtration rate, MTF: metformin, SU: sulfonylurea, TZD: Thiazolidinedione, DPP4i: Dipeptidyl peptidase-4 inhibitor, ACA: acarbose, , EXE: Exenatide. SGLT: SGLT2i, GLP1: Glucagon-like peptide-1 receptor anonist, IPW: Inverse Probability Weight, FU: Follow-up, SD: Standard deviation, ARF: Acute Renal Failure, CKD: Chronic Kidney Disease, IQR: Inter Quartile Range, p-yr: person-years, NR: Not reported, DB: Database, KDIGO: Kidney Disease: Improving Global Outcomes. One further comparison not included here. Hung *et al.* 2012, as two studies by Hung *et al.* reported similar comparison using similar data

### Monotherapy comparisons

***Metformin monotherapy vs. thiazolidinedione monotherapy.*** The most common drug comparison was metformin monotherapy vs. thiazolidinedione monotherapy (five studies made seven comparisons) ^14, 16, 19, 22, 23^. Two RCTs found that thiazolidinediones were associated with improved kidney outcomes (reduced proteinuria or improved eGFR) compared to metformin ^14, 16^ while two observational studies found no differences between the two drug classes ^19, 22^. One observational cohort study showed that thiazolidinediones were associated with a higher risk for development of kidney failure (a composite of kidney dialysis, kidney transplant and CKD stage five) compared to metformin ^23^.

***Metformin monotherapy vs. sulfonylurea monotherapy.*** Six observational studies ^19– 23, 26^ compared metformin monotherapy to sulfonylurea monotherapy. Though two of these studies ( 19 and 20) reported similar findings from the same source population, we have therefore only reported one of the results, making six comparisons. Four comparisons favoured metformin. One study found the risk of eGFR falling to below 60 mL/min/1.73m ^2^ was greater in the sulfonylurea group compared to the metformin group ^22^. Three found higher risks of kidney failure outcomes (various composites of codes for nephropathy, dialysis, renal transplant, ESRD, and reductions in eGFR) for sulfonylurea compared to metformin ^20, 21, 23^. One study, using proteinuria as an outcome, found no difference between drug classes ^22^. One further study reported higher rates of acute dialysis for people initiating metformin compared to sulfonylureas ^26^.

***Sulfonylurea monotherapy vs. thiazolidinedione monotherapy.*** Findings from two RCTs showed differences in ACR that were not statistically significant ^12, 16^. However, one of these studies also showed an increase in mean eGFR among patients treated with a TZD, but a fall in the SU group ^16^.

***Sulfonylurea monotherapy vs. SGLT2i monotherapy.*** One RCT showed canagliflozin slowed kidney function decline, and reduced albuminuria, compared to glimepiride ^17^.

### Combination therapy comparisons

Only three studies compared combination therapies.

***Metformin plus sulfonylurea vs. metformin plus thiazolidinedione.*** One RCT compared metformin plus sulfonylurea to metformin plus a thiazolidinedione ^15^. They reported that ACR decreased in the metformin plus thiazolidinedione group and increased in the metformin plus sulfonylurea group ^15^.

***Sulfonylurea plus metformin vs. sulfonylurea plus thiazolidinedione.*** One RCT compared sulfonylurea plus metformin to sulfonylurea plus thiazolidinedione ^13^. The study found that the ACR increased in the sulfonylurea plus metformin group, and decreased in the sulfonylurea plus thiazolidinedione group ^13^.

***Metformin plus sulfonylurea vs. metformin plus gliptin (DPP4i).*** One observational study compared metformin plus sulfonylurea combination therapy to metformin plus sitagliptin ^25^. The results showed weak evidence that metformin plus sitagliptin improved the likelihood of reductions in ACR, with an odds ratio of 1.20 (95% CI: 0.99–1.47, P = 0.063) ^25^.

### Dual therapy vs. monotherapy

Three observational studies made seven comparisons between monotherapy options and combination therapy ^20, 21, 23^. One study indicated that people taking metformin were at a lower risk of renal failure compared to people taking metformin plus sulfonylurea ^21^. Another study found the opposite, people taking metformin plus sulfonylurea were at lower risk of kidney failure compared to metformin ^23^. The same study found no differences in the risk of kidney failure compared to metformin in people prescribed; i) metformin plus thiazolidinedione, and ii) metformin plus gliptin. They also reported that people prescribed sulfonylurea plus thiazolidinedione, and a sulfonylurea plus DPP4i were at higher risk for kidney failure compared to metformin ^23^.

Another observational study found no difference in eGFR outcomes between sulfonylurea monotherapy and metformin plus sulfonylurea combination therapy ^20^.

### Study quality

We assessed each study for quality, using the GRACE 2014 ^10^ items for observational comparative effectiveness research and the Cochrane Collaboration risk of bias tool for RCTs ^11^ Supplementary Table 5 and Supplementary Table 6 ( Supplementary File 2) detail the results. For the RCTs, we assessed study quality as good, though few studies reported details of randomisation techniques. Of the observational studies, reporting was reasonable, according to the GRACE criteria. However, many of the studies made comparisons between drugs used at different stages of drug intensification, or between monotherapy and combination therapy. For example, two observational studies ^21, 23^ used metformin monotherapy as the baseline in comparisons with combination therapy. As metformin monotherapy is the most common drug for initiating treatment, and the addition of other drugs to metformin is likely to be associated with progression or poor control of type 2 DM, comparing metformin to drug prescribed at the first stage of intensification is problematic, particularly for renal outcomes. Those people receiving treatment intensification will tend to be sicker, and distinguishing between the effects of treatment and the effects of the underlying disease may not always be possible.

## Conclusion

### Key findings

Overall, we have found a lack of consistent evidence of long-term differences in kidney outcomes between T2DM drugs. In comparisons of treatments for type 2 DM, for thiazolidinediones vs metformin, there is some evidence of reduced proteinuria - of four comparisons with ACR as an outcome (in combination or monotherapy), three favoured TZD and one showed no difference. Most evidence from observational research also suggested that metformin is associated with better kidney outcomes than sulfonylureas.

Despite frequent use of combination therapies for the treatment of diabetes, we found few studies that compared commonly used dual therapies that investigated renal outcomes.

### Previous work

The finding that thiazolidinediones may reduce proteinuria compared with metformin is aligned with observations of other authors and supported by animal studies ^27, 28^. Though previous evidence is limited, other work suggests that TZDs could exert reno-protective effects via a number of pathways, including reducing blood pressure ^28^. TZDs may also act directly in the kidneys via proliferator-activated receptor gamma (PPARg), found in the kidney (and in other tissue) ^27, 28^. However, changes in estimated GFR may reflect changes in fluid status rather than true changes in renal function, which was not measured directly in any study ^29^.

### Strengths

To our knowledge, this is the first systematic review of the comparative research literature that investigated the effects of type 2 diabetes drug regimens on renal function. We have conducted an extensive and detailed search, with broad definitions of renal function.

### Limitations

We have focused on renal outcomes only but recognize this is just one of many safety and effectiveness factors to be considered when deciding treatment options. Despite the importance of careful monitoring and maintenance of kidney function for people with diabetes, we identified just 15 long-term studies reporting renal outcomes. Renal complications of type 2 diabetes take many years to develop after the onset of diabetes and studies may not be adequately powered or have sufficient length of follow-up to detect differences. Therefore, many studies have used the surrogate marker of changes in proteinuria as a marker of clinical renal outcomes. Further, initial changes in kidney function may be misleading. One included study indicates benefits of canagliflozin over glimipiride for kidney function decline at 104 weeks: however these benefits were not apparent until 52 weeks ^17, 30^. This and the EMPA-REG study ^31^ have indicated initial acute falls in eGFR with better outcomes compared to placebo only observed over the longer term so this would not be apparent in short-term studies.

Our review included both randomised and non-interventional studies. Whilst the unique inferential advantages of randomization are clear, our review highlights a large overall difference in population size depending on study type: randomised trials generally included hundreds of patients, whilst non-interventional studies often had tens of thousands of participants. Rarer outcomes such as ESRD are therefore more likely to be detected in non-interventional settings. This highlights their important role, but the evidence generated from them needs to be evaluated cautiously due to the potential for bias and confounding.

The available evidence does not reflect drugs currently prescribed in routine care. In our review, 69% (22/32) of the comparisons, contrasted different monotherapies, with just three comparisons between dual therapy combinations. In clinical practice, metformin is the most common first-line therapy, and GPs now rarely prescribe thiazolidinediones (EU marketing authorization for Rosiglitazone was suspended in 2010 ^32^, following concern regarding increased heart failure risk) ^33^.

In the UK, NICE guidance recommends the addition of sulfonylureas, Dipeptidyl peptidase-4 inhibitors (DPP4is) Sodium-glucose Cotransporter 2 Inhibitors (SGLT2is), or TZDs to metformin, yet, just one study compared these combinations (MTF+SU vs MTF+DPP4i) ^25, 33– 35^. Recent studies that have shown potentially exciting improvements in renal outcomes for patients treated with SGLT2is were conducted against placebo and so were not eligible for this study ^36, 37^.

We found that definitions of kidney outcomes were not consistent across studies. Definitions of renal decline in the observational studies relied upon either codes for kidney disease (e.g. diabetic nephropathy, acute renal failure), surrogate markers (e.g. eGFR or proteinuria) or a combination of codes and tests, summarised in Supplementary Table 4 ( Supplementary File 2). For the albuminuria data, which has a skewed distribution, most studies used logarithmic transformation to approximate normal, yet not all studies applied this method ^18^. Such differences between outcomes will limit future opportunities for pooling effect estimates in meta-analyses. Different approaches to study design may also limit the validity of findings. We found two observational studies that made the same comparisons yet found different effects. Both examined renal failure, using UK primary care data, (QResearch ^23^ and Clinical Practice Research Datalink ^21^). They found comparable effect sizes when comparing the use of sulfonylurea monotherapy to metformin monotherapy, for renal failure (2.63, 95% CI: 2.25, 3.06 ^23^ and 2.63, 95% CI: 2.19, 3.15 ^21^). However, when comparing sulfonylurea plus metformin dual therapy to metformin monotherapy, estimates of the risk of kidney failure were in opposite directions (0.76, 95% CI: 0.62, 0.92 ^23^ and 1.39, 95% CI: 1.12, 1.72 ^21^). Difficulties in adjusting for levels of diabetic control or change in renal function that led to these treatment choices (confounding by indication), may explain these conflicting results.

In the randomised controlled studies, we found that eligibility criteria were strict. Many studies excluded people most at risk of kidney outcomes e.g. those with reduced kidney function or cardiovascular disease ^12, 13, 15– 18^. These restrictions limit the generalisability of study findings to routine clinical settings where people presenting with diabetes have complex comorbidities ^38^. Further, as most individuals with type 2 diabetes will receive treatment for other comorbid conditions, prescribers need to know how diabetic therapies interact with concomitant drugs, yet this is not addressed by the studies identified in this review.

### Clinical relevance

In clinical practice, kidney function is one of many considerations for treatment choice in type 2 DM. Some of the differences we found for albuminuria and eGFR between people taking different oral therapies for type 2 diabetes were statistically significant, but the clinical importance of these findings may be limited. Some surrogate outcomes such as a doubling of creatinine or 30% decline in eGFR are closely associated with risk of future ESRD ^39, 40^ while ACR is not ^39, 41, 42^. Outcomes that are clinically relevant need to be assessed in future studies. Ideally, these should include hard outcomes such as hospital admission with acute kidney injury or the development of ESRD. Therefore, large, well-designed studies with long follow up, including individuals that represent the typical type 2 diabetes population, will be required. However, the incidence of kidney outcomes is likely to be low in most randomised trials and therefore high-quality observational studies will also be needed.

Our review highlights a lack of rigorous studies comparing the effects of oral type 2 diabetes drugs on kidney outcomes, in particular, for the newer drug intensification options where prescribing is rapidly increasing.

### Data availability

All data underlying the results are available as part of the article and supplementary material no additional source data are required.

## Supplementary material

Supplementary File 1 – Completed PRISMA checklist

Supplementary File 2 – File contain the following supplementary tables.

Supplementary Table 1: First Ovid Medline search

Supplementary Table 2: First search Web of science

Supplementary Table 3: Report of further comparisons from Hippisley-Cox and Coupland (2016) paper

Supplementary Table 4: Detailed definitions of composite renal outcomes for observational studies

Supplementary Table 5: GRACE 2014 items for observational studies

Supplementary Table 6: Cochrane items for quality of RCT studies
