# Supplementary material for: A systematic review comparing the evidence for kidney function outcomes between oral antidiabetic drugs for type 2 diabetes
Source: Wellcome Open Res. 2018 Jun 19;3:74. [Version 1] doi: 10.12688/wellcomeopenres.14660.1 (PMC6107985; doi:10.12688/wellcomeopenres.14660.1)
Supplement: Supplementary file 2 [file wellcomeopenres-3-15962-s0001.tgz › 8d572a2c-9b11-4678-9f2e-4a967385ecee.docx]

# Supplementary information

### Table 1: First Ovid Medline search

| 1 | (kidney or renal or Albumin or ACR or albuminuria or CKD or creatinine or dialysis or eGFR or esrd or glomerular or GFR* or cystatinC or haematuria or microvascular or protein:creatinine or proteinurea or proteinuria).af. | 1152518 | Advanced |
| --- | --- | --- | --- |
| 2 | Renal Insufficiency/ or Cystatin C/ or Kidney/ or Kidney Diseases/ or Glomerular Filtration Rate/ or Creatinine/ or Diabetic Nephropathies/ or Proteinuria/ | 389625 | Advanced |
| 3 | 1 or 2 | 1161071 | Advanced |
| 4 | (antidiabetic or Acarbose or Acetohexamide or Actos or Actraphane or Alogliptin or glucosidase or Amaryl or Aspart or Avandia or Avandamet or insulin or Biguanide* or Bolamyn or Bydureon or Byetta or Calabren or Canagliflozin or Chloropropamide or Competact or Dacadis or Daonil or Dapagliflozin or Degludec or Determir or Diabetamide or Diabinese or Diagemet or Diaglyk or Diamicron or Dimelor or Dipeptidyl or DPP-4 or DPP-4i or Duclazide or Duformin or Edicil or Empagliflozin or Enyglid or Eucreas or Euglucon or Exenatide or Forxiga or Galvus or Glargine or Glibenclamide or Glibenese or Glibornuride or Gliclazide or Gliflozin or Glimepiride or Glinides or Glipizide or Gliquidone or Glitpins or GLP-1 or Glucagon-like or Glucamet or Glucient or Glucobay or Glucophage or Glutril or Glyconon or Glymese or Glymidine or Guar or Guarem or Guarina or Glurenorm or Humaject or Humalog or Humulin or Hypurin or Hypurin or incretin or Innolet or Insulatard or Insulin or Invokana or aspart or degludec or detemir or glargine or Isophane or Lispro or Insuman or Invokana or Isophane or Janumet or Januvia or Jentadueto or Komboglyze or Laaglyda or Lantus or Levemir or Libanil or Linagliptin or Liraglutide or Lisophane or Lispro or Lixisenatide or Lyxumia or Malix or Meglinitides or Metabet or Metformin or Minodiab or Dapagliflozin or Pioglitazone or Saxagliptin or Vildagliptin or Metsol or Mixtard or Nateglinide or Nateglinide or Nazdol or Neuphane or Niddaryl or Novomix or NovoNorm or Onglyza or Orabet or Penmix or Pioglitazone or Prandin or Protamine or Pur-in or Rastinon or Repaglinide or Romozin or Rosiglitazone or Maleate or Saxagliptin or Semi-Daonil or Sitagliptin or Starlix or SU or Sukkarto or Sulfonylurea or Sulphonylurea or Tempulin or Tolanase or Tolazamide or Tolbutamide or Trajenta or Tresiba or Troglitazone or TZD or thiazolidinedione or Victoza or Vildagliptin or Vildagliptin or Vipdomet or Vipidia or Xigduo or Zicron).ab,ti. | 332666 | Advanced |
| 5 | Hypoglycemic Agents/ | 48689 | Advanced |
| 6 | 4 or 5 | 346663 | Advanced |
| 7 | (Case-control* or Cohort* or Comparative effectiveness* or Cross-sectional or Meta-analysis or Nonexperimental or Pharmacoepid* or Prospectiv* or RCT* or Trial*).ab,ti. | 1487119 | Advanced |
| 8 | Clinical Trial/ or Comparative Effectiveness Research/ or Cohort Studies/ or Cross-Sectional Studies/ or Case-Control Studies/ | 1063484 | Advanced |
| 9 | 7 or 8 | 2064294 | Advanced |
| 10 | 3 and 6 and 9 | 6491 | Advanced |
| 11 | limit 10 to (male and female and humans and yr="1980 -Current") | 4083 | Advanced |

**Table 1** First Ovid Medline search

### Table 2 First search Web of science

| **Set** | **Results** | **Criteria** |
| --- | --- | --- |
| # 4 | 532 | #3 AND #2 AND #1  *Timespan=1980-2016*  *Search language=Auto* |
| # 3 | [Approx.](https://apps.webofknowledge.com/summary.do?product=UA&doc=1&qid=4&SID=2AfpM9JWVEOsrYU1QTw&search_mode=AdvancedSearch&update_back2search_link_param=yes)  [559,338](https://apps.webofknowledge.com/summary.do?product=UA&doc=1&qid=4&SID=2AfpM9JWVEOsrYU1QTw&search_mode=AdvancedSearch&update_back2search_link_param=yes) | TI=(antidiabetic or Acarbose or Acetohexamide or Actos or Actraphane or Alogliptin or glucosidase or Amaryl or Aspart or Avandia or Avandamet or insulin or Biguanide* or Bolamyn or Bydureon or Byetta or Calabren or Canagliflozin or Chloropropamide or Competact or Dacadis or Daonil or Dapagliflozin or Degludec or Determir or Diabetamide or Diabinese or Diagemet or Diaglyk or Diamicron or Dimelor or Dipeptidyl or "DPP-4" or "DPP-4i" or Duclazide or Duformin or Edicil or Empagliflozin or Enyglid or Eucreas or Euglucon or Exenatide or Forxiga or Galvus or Glargine or Glibenclamide or Glibenese or Glibornuride or Gliclazide or Gliflozin or Glimepiride or Glinides or Glipizide or Gliquidone or Glitpins or "GLP-1" or Glucagon-like or Glucamet or Glucient or Glucobay or Glucophage or Glutril or Glyconon or Glymese or Glymidine or Guar or Guarem or Guarina or Glurenorm or Humaject or Humalog or Humulin or Hypurin or Hypurin or incretin or Innolet or Insulatard or Insulin or Invokana or aspart or degludec or detemir or glargine or Isophane or Lispro or Insuman or Invokana or Isophane or Janumet or Januvia or Jentadueto or Komboglyze or Laaglyda or Lantus or Levemir or Libanil or Linagliptin or Liraglutide or Lisophane or Lispro or Lixisenatide or Lyxumia or Malix or Meglinitides or Metabet or Metformin or Minodiab or Dapagliflozin or Pioglitazone or Saxagliptin or Vildagliptin or Metsol or Mixtard or Nateglinide or Nateglinide or Nazdol or Neuphane or Niddaryl or Novomix or NovoNorm or Onglyza or Orabet or Penmix or Pioglitazone or Prandin or Protamine or Pur or Rastinon or Repaglinide or Romozin or Rosiglitazone or Maleate or Saxagliptin or Daonil or Sitagliptin or Starlix or SU or Sukkarto or Sulfonylurea or Sulphonylurea or Tempulin or Tolanase or Tolazamide or Tolbutamide or Trajenta or Tresiba or Troglitazone or TZD or thiazolidinedione or Victoza or Vildagliptin or Vildagliptin or Vipdomet or Vipidia or Xigduo or Zicron or Hypoglycaemic or Hypoglycemic)  *Timespan=1980-2016*  *Search language=Auto* |
| # 2 | [Approx. 1,428,406](https://apps.webofknowledge.com/summary.do?product=UA&doc=1&qid=3&SID=2AfpM9JWVEOsrYU1QTw&search_mode=AdvancedSearch&update_back2search_link_param=yes) | TI=(Cohort* or Comparative or Nonexperimental or Pharmacoepid* or Prospectiv* or RCT* or Trial*)  *Timespan=1980-2016*  *Search language=Auto* |
| # 1 | [Approx. 2,999,026](https://apps.webofknowledge.com/summary.do?product=UA&doc=1&qid=1&SID=2AfpM9JWVEOsrYU1QTw&search_mode=AdvancedSearch&update_back2search_link_param=yes) | TS=(kidney or renal or Albumin or ACR or albuminuria or CKD or creatinine or dialysis or eGFR or esrd or glomerular or GFR* or cystatinC or haematuria or microvascular or proteinurea)  *Timespan=1980-2016*  *Search language=Auto* |

**Table 2** First search Web of science, across Web of ScienceTM Core Collection, BIOSIS Citation Index, KCI-Korean Journal Database, MEDLINE, SciELO Citation Index

### Table 3 Report of further comparisons from Hippisley-Cox and Coupland (2016) paper

| **Author  (Year)** | **Kidney outcomes** | **Kidney outcomes recorded**  **HR (95% CI)** |
| --- | --- | --- |
|  |  |  |
| Hippisley-Cox and Coupland (2016) (19) | **1** Incident severe kidney failure (Read code defined as kidney dialysis, transplantation, or CKD stage 5 based on serum creatinine values | Incident kidney failure  MTF referent  TZD: 2.55 (1.13, 5.74)  DPP4I: 3.52 (2.04, 6.07)  SU: 2.63 (2.25, 3.06)  MTF+TZD: 0.71 (0.33, 1.50)  MTF+SU: 0.76 (0.62, 0.92)  MTF+DPP4I: 0.59 (0.28, 1.25)  SU+TZD: 2.14 (1.27, 3.61)  SU+DPP4I: 3.21 (2.08, 4.93)  SU+TZD+MTF: 1.21 (0.75, 1.96)  SU+DPP4I+MTF: 0.68 (0.39, 1.20) |
| **Abbreviations**: MTF: metformin, SU: sulfonylurea, TZD: thiazolidinedione, DPP4I: Dipeptidyl peptidase-4, CKD: Chronic Kidney Disease | | |

**Table 3** Report of further comparisons from Hippisley-Cox and Coupland (2016) paper

### Table 4 Detailed definitions of composite renal outcomes for observational studies

| **Author (Year)** | **Definition of Renal outcomes** |
| --- | --- |
| Hung et al (2012) (20) | 1: eGFR event  >=25% decline, confirmed 3-12 months following  2: ESRD  Defined as eGFR<15 (confirmed in 3-12 months following), ICD-9 codes for dialysis (confirmed in 3-12 months following), renal transplant  3: Mortality |
| Pendergrass et al (2012) (24) | 1st ARF (ICD-9 code 584*) |
| Currie et al. (2013) (17) | Renal failure (Read code defined, covering disease areas: CKD, dialysis, transplantation, renal failure, nephritis, nephropathy, necrosis) |
| Hung et al (2013) (21) | 1: eGFR event  >=25% decline, confirmed 3-12 months following  2: ESRD  Defined as eGFR<15 (confirmed in 3-12 months following), ICD-9 codes for dialysis (confirmed in 3-12 months following), renal transplant  3: Mortality |
| Masica et al. (2013) (23) | 1: New proteinuria (based on 24-hour albumin/protein, spot protein, spot ACR, or dipstick)  2: New eGFR to <60 |
| Hippisley-Cox and Coupland (2016) (19) | 1: Incident severe kidney failure (Read code defined as kidney dialysis, transplantation, or CKD stage 5 based on serum creatinine values) |
| Kolaczynski et al (2016) (22) | Incident nephropathy (ICD-10 codes: E11.2 or E14.2) |
| Goldshtein et al. (2016) (18) | Improvements in urinary ACR (at least 20% improvement in ACR and change in KDIGO category) |
| **Abbreviations**: ESRD: End stage renal disease, ARF: Acute Renal Failure, ACR: Albumin: Creatinine Ratio, CKD: Chronic Kidney Disease, KDIGO: Kidney Disease: Improving Global Outcomes, ICD: International Classification of Diseases | |

**Table 4** Detailed definitions of composite renal outcomes for observational studies

### Table 5 GRACE 2014 items for observational studies

|  | Authors, year **Scoring as Fit for Purpose: Sufficient (+), Insufficient (-)** | | | | | | | |
| --- | --- | --- | --- | --- | --- | --- | --- | --- |
| **Component Item** | **Hung 2013** | **Hung 2012** | **Currie et al. 2013** | **Masica et al. 2013** | **Kolaczynski et al. 2016** | **Hippisley-Cox & Coupland, 2016** | **Goldshtein et al. 2016** | **Carlson et al 2016** |
| **Data** | | | | | | | | |
| D1. Were treatment and/or important details of treatment exposure adequately recorded for the study purpose in the data source(s)? | + | + | + | - | - | + | + | + |
| D2. Were the primary outcomes adequately recorded for the study purpose (e.g., available in sufficient detail through data sources)? | + | + | + | + | + | + | + | + |
| D3. Was the primary clinical outcome(s) measured objectively rather than subject to clinical judgment (e.g., opinion about whether the patient’s condition has improved)? | + | + | + | + | + | + | + | + |
| D4. Were primary outcomes validated, adjudicated, or otherwise known to be valid in a similar population? | + | + | + | + | - | + | + | + |
| D5. Was the primary outcome(s) measured or identified in an equivalent manner between the treatment/intervention group and the comparison group? | + | + | + | + | + | + | + | + |
| D6. Were important covariates that may be known confounders or effect modifiers available and recorded? | + | + | + | + | - | + | + | + |
| **Methods** | | | | | | | | |
| M1. Was the study (or analysis) population restricted to new initiators of treatment or those starting a new course of treatment? | ? | + | - | - | - | - | + | + |
| M2. If 1 or more comparison groups were used, were they concurrent comparators? | + | + | + | + | + | + | + | + |
| M3. Were important confounding and effect-modifying variables taken into account in the design and/or analysis? | + | + | + | + | + | - | + | + |
| M4. Is the classification of exposed and unexposed person-time free of “immortal time bias? | + | + | + | + | + | + | + | + |
| M5. Were any meaningful analyses conducted to test key assumptions on which primary results are based? | + | + | some | - | - | - | + | + |

**Table 5** GRACE 2014 items for observational studies

### Table 6 Cochrane items for quality of RCT studies

|  | Authors, year  **Scoring as Fit for Purpose: Sufficient (+), Insufficient (-)** | | | | | | |
| --- | --- | --- | --- | --- | --- | --- | --- |
| **Item** | **Bakris, 2003** | **Hanefeld, 2004** | **Schernthaner et al. (2004)** | **Matthews, 2005** | **Lachin, 2011** | **Heerspink et al. 2017** | **Pan et al. 2016** |
| Random sequence generation (selection bias) | ? | ? | + | ? | ? | + | - |
| Allocation concealment (selection bias) | - | ? | + | ? | ? | + | - |
| Blinding of participants and researchers (performance bias) | - | + | + | + | + | + | - |
| Blinding of outcome assessment (detection bias) | ? | + | ? | + | + | + | - |
| Incomplete outcome data (attrition bias) | ? | + | ? | ? | - | + | ? |
| Selective reporting (reporting bias) | + | + | + | + | + | + | + |

**Table 6** Cochrane items for quality of RCT studies
