# Supplementary material for: A systematic review comparing the evidence for kidney function outcomes between oral antidiabetic drugs for type 2 diabetes
Source: Wellcome Open Res. 2018 Jun 19;3:74. [Version 1] doi: 10.12688/wellcomeopenres.14660.1 (PMC6107985; doi:10.12688/wellcomeopenres.14660.1)
Supplement: Peer review history

# Peer review: Referee response for version 1

**DOI:** 10.21956/wellcomeopenres.15962.r33480

**Published:** 2018-08-17

**Authors:** Christian Fynbo Christiansen, Søren Viborg Vestergaard

**Affiliations:** 1 Department of Clinical Epidemiology, Aarhus University Hospital, Aarhus, Denmark

We have read the paper by Wilkinson *et al*. with great interest. The paper reports a systematic literature review of studies examining the kidney prognosis in patients treated with different combinations of antidiabetic drugs in Type II diabetes. The study found a lack of literature to draw firm conclusions.

 The topic is important, and the paper is well written and follows the PRISMA guidelines. The paper describes the elements of the search strategy and the authors reviewed an extensive amount of papers to end up with a small sample of relevant papers. Due to substantial variety in kidney function outcomes and drug class comparisons, the authors did not conduct a meta-analysis.

 We have only a few comments to the article:

1. Potential uncontrolled confounding by indication (and contraindication) are probably the most important limitation when interpreting the findings of the included observational studies. In particular, because metformin is the recommended first-line treatment in patients without renal impairment. It could be more clear whether the estimates included in Table 1 “ *kidney outcomes recorded HR*” are adjusted for relevant confounders and what confounders that were included in each study.
2. Figure 2 is very illustrative and a good way to summarize data in this review. Unfortunately, it is not possible to see the strength of the associations in such a figure. Would it be possible to use different line thickness to illustrate the strength of the associations?
3. The introduction states that the study focuses on “ *following outcomes: change in kidney function (estimated glomerular filtration rate), progression or development of proteinuria, development of end-stage renal disease (ESRD) and composite outcomes”* (page 3). However in the result section following outcomes are mentioned “ *changes in eGFR […] albumin-creatinine ratio (ACR) […] kidney endpoints, including kidney failure, nephropathy, acute dialysis and composite endpoints with eGFR” *(page 3) *.* Finally, in Table 3 the studies are divided in the three groups “ *ACR, eGFR, and Kidney outcomes” *based on the study endpoints (Table 3). We suggest that the terms describing other kidney outcomes than ACR and eGRF are clearly defined and used consequently throughout the paper.
4. It is not clear, whether the final search strings differed substantially from the first searches, which are described in supplementary Table 1 and 2.

We have read this submission. We believe that we have an appropriate level of expertise to confirm that it is of an acceptable scientific standard.

# Peer review: Referee response for version 1

**DOI:** 10.21956/wellcomeopenres.15962.r33343

**Published:** 2018-06-25

**Authors:** William G. Herrington

**Affiliations:** 1 Clinical Trial Service Unit and Epidemiological Studies Unit (CTSU), University of Oxford, Oxford, UK; 2 Medical Research Council Population Health Research Unit, University of Oxford, Oxford, UK

I like the approach to article screening by random checking rather than duplicating reviewer work in its entirety. This could be risk-based in future reviews.

 My only comments relate to the discussion:

 1. The authors state “most evidence from observational research also suggested that metformin is associated with better kidney outcomes than sulphonylureas”. Indirect comparison could be a good sanity check that this is as expected. For example, do the placebo-controlled trials show that metformin has beneficial effects on kidney outcomes and do placebo-controlled trials of sulphonylureas predict they may differ? 

 2. The penultimate paragraph concludes that: “….high-quality observational studies are needed” to address the effect of different antidiabetes drugs on ESRD or hospitalization with acute kidney injury. As the authors acknowledge, such studies require careful adjustment for confounders. The particular challenges this poses in populations with type 2 diabetes could be more clearly highlighted in the discussion. First, co-morbidity and co-medication are common, which increases the number of covariates required for reliable findings to emerge. Secondly, complete and precise measurement of all relevant confounders are difficult to ensure. For example, HbA1c, BP and RAS-inhibition use throughout the observation period (and arguably in the period which precedes it) would all be important to consider adjusting for, but measurement error is common for these parameters and defining and using covariates can be problematic (e.g. differences in RAS-inhibition formulations, doses and adherence).

I have read this submission. I believe that I have an appropriate level of expertise to confirm that it is of an acceptable scientific standard.
